# Stakeholder Criteria for Trust in Artificial Intelligence–Based Computer Perception Tools in Health Care: Qualitative Interview Study

**DOI:** 10.2196/78757

**Published:** 2025-12-12

**Authors:** Ansh Rai, Meghan E Hurley, John Herrington, Eric A Storch, Casey J Zampella, Julia Parrish-Morris, Anika Sonig, Gabriel Lázaro-Muñoz, Kristin Kostick-Quenet

**Affiliations:** 1 Center for Medical Ethics and Health Policy Baylor College of Medicine Houston United States; 2 Children's Hospital of Philadelphia Philadelphia United States; 3 Department of Psychiatry and Behavioral Sciences Baylor College of Medicine Houston, TX United States; 4 Center for Bioethics Harvard Medical School Boston, MA United States; 5 Department of Psychiatry and Behavioral Sciences Massachusetts General Hospital Boston, MA United States

**Keywords:** artificial intelligence, computing, trustworthiness, trust, technology, machine learning, psychiatry

## Abstract

**Background:**

Computer perception (CP) technologies hold significant promise for advancing precision mental health care systems, given their ability to leverage algorithmic analysis of continuous, passive sensing data from wearables and smartphones (eg, behavioral activity, geolocation, vocal features, and ambient environmental data) to infer clinically meaningful behavioral and physiological states. However, successful implementation critically depends on cultivating well-founded stakeholder trust.

**Objective:**

This study aims to investigate, across adolescents, caregivers, clinicians, and developers, the contingencies under which CP technologies are deemed trustworthy in health care.

**Methods:**

We conducted 80 semistructured interviews with a purposive sample of adolescents (n=20) diagnosed with autism, Tourette syndrome, anxiety, obsessive-compulsive disorder, or attention-deficit/hyperactivity disorder and their caregivers (n=20); practicing clinicians across psychiatry, psychology, and pediatrics (n=20); and CP system developers (n=20). Interview transcripts were coded by 2 independent coders and analyzed using multistage, inductive thematic content analysis to identify prominent themes.

**Results:**

Across stakeholder groups, 5 core criteria emerged as prerequisites for trust in CP outputs: (1) epistemic alignment—consistency between system outputs, personal experience, and existing diagnostic frameworks; (2) demonstrable rigor—training on representative data and validation in real-world contexts; (3) explainability—transparent communication of input variables, thresholds, and decision logic; (4) sensitivity to complexity—the capacity to accommodate heterogeneity and comorbidity in symptom expression; and (5) a nonsubstitutive role—technologies must augment, rather than supplant, clinical judgment. A novel and cautionary finding was that epistemic alignment—whether outputs affirmed participants’ preexisting beliefs, diagnostic expectations, or internal states—was a dominant factor in determining whether the tool was perceived as trustworthy. Participants also expressed relational trust, placing confidence in CP systems based on endorsements from respected peers, academic institutions, or regulatory agencies. However, both trust strategies raise significant concerns: confirmation bias may lead users to overvalue outputs that align with their assumptions, while surrogate trust may be misapplied in the absence of robust performance validation.

**Conclusions:**

This study advances empirical understanding of how trust is formed and calibrated around artificial intelligence–based CP technologies. While trust is commonly framed as a function of technical performance, our findings show that it is deeply shaped by cognitive heuristics, social relationships, and alignment with entrenched epistemologies. These dynamics can facilitate intuitive verification but may also constrain the transformative potential of CP systems by reinforcing existing beliefs. To address this, we recommend a dual strategy: (1) embedding CP tools within institutional frameworks that uphold rigorous validation, ethical oversight, and transparent design; and (2) providing clinicians with training and interface designs that support critical appraisal and minimize susceptibility to cognitive bias. Recalibrating trust to reflect actual system capacities—rather than familiarity or endorsement—is essential for ethically sound and clinically meaningful integration of CP technologies.

## Introduction

### Background

Computer perception (CP) technologies—encompassing digital phenotyping, affective computing, and computational behavioral analysis—promise to improve precision health care by offering objective insights into individual behavioral patterns that can help inform diagnosis and personalize treatment. The term “computer perception” refers to the artificial intelligence (AI) subfield of computer “vision,” while also recognizing a broader spectrum and integration of perceptive modalities beyond vision itself (eg, “hearing” via microphones, motion detection via accelerometers). These technologies involve continuous and passive data collection methods using smartphones and wearable devices, and, in rarer cases, implantable sensors such as brain-computer interfaces. Sensor-equipped devices enable clinical researchers to take an “ecological” approach to understanding clinically relevant behavior outside the clinic, providing a diverse array of behavioral metrics—such as geolocation, accelerometry, social interaction, social media activity, and voice patterns—to complement physiological measures such as cardiovascular or neural activity. Given the highly granular and voluminous nature of these data, AI and machine learning algorithms are used to extract clinically relevant inferences about patients’ behaviors and symptoms, with the potential to improve diagnosis, screening, and prognosis across a variety of medical fields.

As CP technologies continue to transition from investigational stages to practical applications, trustworthiness emerges as a critical yet underexplored factor influencing their adoption and effective integration into clinical practice. While numerous studies focus on the trustworthiness of AI tools broadly [1,2], and others examine the trustworthiness of specific applications (eg, smartphone apps) [3,4], this paper uniquely offers empirical findings from an examination of trustworthiness criteria among a sample of clinicians and patients reflecting on the potential integration of CP technologies into patient care. Identifying various stakeholders’ contingencies for trust is critical for understanding the conditions under which end users of these technologies are likely to integrate CP outputs into clinical decision-making (acceptance and uptake), and for assessing whether their perceived trustworthiness is properly calibrated to the current capacities and limitations of existing CP tools.

### Trust Considerations for Computer Perception Technologies

Trust is essential for clinicians, patients, and other stakeholders to embrace CP innovations and fully realize their benefits. However, trust is shaped by a variety of factors and does not always reflect a system’s actual performance parameters. Rather, it reflects what or how users perceive the system to be or to do, relative to an ideal. Trust plays a vital role in scenarios where the outcomes of using a system are uncertain. In line with classic explorations of trust in new technologies, we define trust as “the attitude that an agent will help achieve an individual’s goals in a situation characterized by uncertainty and vulnerability [5].” In their extrapolation of human trust criteria to automated systems, Lee and Moray [6] argue that humans base their attributions of trust on perceptions and observations of system behavior (performance), their understanding of the underlying mechanisms by which a system arrives at conclusions (process), and assessments of the intended use of the system (purpose) [6]. Ideally, users adjust their level of trust in CP technologies based on a thorough appraisal of a system’s performance capacities, including whether it exemplifies certain pillars of trustworthiness (eg, accuracy, reliability, transparency, and explainability) outlined by national [7] and international [8,9] frameworks. However, proper calibration does not characterize every trust relationship, and people can over- or under-trust systems for a variety of reasons. For example, a person’s experience with or disposition toward technology can influence their level of trust [10,11]. Other socioeconomic and structural factors, such as familiarity with and facility in using technology, can also play a role. Research has linked higher AI literacy with more positive and trusting attitudes toward AI [12-14], though some studies have found the opposite, suggesting that greater understanding can, in some contexts, heighten skepticism rather than trust [15]. Contextual and systemic factors, such as regulatory, institutional, and peer influences [16,17], can likewise affect trust. Importantly, the level of trust that people place in these technologies may not align with what is justified by a system’s performance. Scholars [18] and policy makers have thus pointed out the distinction between trust (the level of confidence users place in a system) and trustworthiness (the features that render a system worthy of being trusted).

Further, given that CP technologies are a subset of AI applications in health care, they inherit many of the same trust issues seen in broader AI deployments. The algorithms that enable inferences from CP tools (process) are intended (purpose) to offer insights into complex conditions that are difficult to observe or measure directly, such as mental health conditions or subtle psychosocial, behavioral, or physiological changes [19-21]. In psychiatry—where CP has been increasingly examined—CP technologies offer tools to analyze behavioral patterns and physiological signals that may not be readily apparent in traditional clinical assessments [22]. However, the heterogeneity among psychiatric presentations makes classification and diagnosis (performance) inherently challenging [23,24]. The intricacy of data patterns that often give rise to diagnostic or prognostic inferences raises concerns about transparency, explainability, and the potential for overreliance on technology at the expense of human judgment. The opacity of certain algorithms can potentially lead to skepticism or, conversely, unwarranted over-trust in the technology’s outputs (eg, due to algorithmic bias). Effective and responsible integration of these technologies into clinical practice, therefore, requires clinicians and other stakeholders to consider multiple system-level features when evaluating the trustworthiness of CP tools and to calibrate their trust accordingly.

Consideration of other ethical concerns, such as privacy and data protection, also influences the trustworthiness of integrating CP into care [25]. CP tools entail passive, continuous collection of often sensitive data via patients’ daily interactions with smartphones, wearables, and other devices, raising concerns about informed consent, as patients may not be fully aware of the extent or nature of the data being collected or how these data may be used to enable both clinical and nonclinical inferences [26,27]. The collection of such potentially sensitive data also introduces ethical dilemmas related to data security risks and the potential misuse of patient information, which can impact patient autonomy and trust in health care systems [28].

Finally, there are concerns about the potential for data-driven CP technologies to displace patient perspectives as a primary source of insight into their conditions [29]. Clinical evaluations and treatment decisions that are highly mediated by data and technology risk overshadowing important qualitative aspects of patient care [22], potentially leading to a diminished role for patient self-reporting, autonomy, and subjective experiences.

To explore the criteria under which trustworthiness might be established for CP technologies in precision mental health systems, we conducted interviews with a diverse set of stakeholders involved in the development, future deployment, or use of CP tools. To effectively adopt the distinctions made in recent literature between trust and trustworthiness in AI systems, our interviewees’ trust responses should be interpreted as user attitudes that may subjectively track a tool’s trustworthiness. Our findings are intended to advance empirical understandings of how trustworthiness is formed around AI-based CP technologies and to ensure that trustworthiness is calibrated to properly reflect actual system capacities for ethically sound and clinically meaningful integration into care.

## Methods

### Design

As part of a 4-year National Institutes of Health–funded study (R01TR004243), we conducted in-depth, semistructured interviews (total N=80) with adolescent patient (n=20) and caregiver (n=20) dyads, clinicians (n=20), and CP developers (n=20) to explore their perspectives on the potential benefits, risks, and concerns surrounding the integration of CP technologies into clinical care. See Multimedia Appendix 1 for the COREQ (Consolidated Criteria for Reporting Qualitative Research) checklist.

### Participants

Adolescent participants ranged in age from 12 to 17 years. Diagnostic presentations for all adolescents were confirmed by expert clinicians using a battery of well-established clinical measures. Caregivers were typically biological parents (Table 1). Adolescent-caregiver dyads were referred to this study by a “sister study” (R01MH125958) and then contacted by a research assistant (AS) via phone or email to schedule an interview. Clinicians included practicing psychiatrists, psychologists, and pediatricians. Clinicians and developers were identified through existing professional networks and online literature searches and were contacted by a research assistant (MEH) via email to schedule an interview. Clinical specialties ranged across psychiatry (7/20, 35%), psychology (7/20, 35%), neuroscience (4/20, 20%), and other fields (2/20, 10%). Developers included senior researchers in academia (3/21, 14%), industry (15/21, 71%), or both (3/21, 14%), each actively working on developing or refining tools intended for passive collection of health-related data or for diagnosis/classification, prediction, or treatment of pathology. Our response rate was approximately 63% for patients and caregivers (63 were contacted, and 40 agreed to participate) and about 41% for clinicians and developers (98 were contacted, and 40 agreed to participate). Decliners cited reasons such as lack of time, lack of interest, or insufficient topical expertise to inform the study goals.

**Table 1:** Demographics for interviewed adolescents and caregivers.

Demographics			Adolescents (n=20)		Caregivers (n=20)		Total (N=40)
**Gender, n (%)** ^a^							
	Male	12 (60)		2 (10)		14 (35)	
	Female	8 (40)		18 (90)		26 (65)	
**Ethnicity, n (%)**							
	American Indian or Alaska Native	0 (0)		1 (5)		1 (3)	
	Asian	1 (5)		1 (5)		2 (5)	
	African American/Black Native Hawaiian or Other	5 (25)		4 (20)		9 (23)	
	Pacific Islander	0 (0)		0 (0)		0 (0)	
	White	17 (85)		15 (75)		32 (80)	
	Hispanic or Latino	4 (20)		2 (10)		6 (15)	
	Not Hispanic or Latino	16 (80)		18 (90)		34 (85)	
**Marital status, n (%)**							
	Married and living with spouse	N/A^b^		13 (65)		13 (33)	
	Widowed	N/A		1 (5)		1 (3)	
	Divorced	N/A		4 (20)		4 (10)	
	Separated	N/A		1 (5)		1 (3)	
	Never married	N/A		1 (5)		1 (3)	
**Education, n (%)**							
	High school only or less	N/A		0 (0)		0 (0)	
	Trade school/associate’s degree	N/A		2 (10)		2 (5)	
	Bachelor’s degree	N/A		10 (50)		10 (25)	
	Master’s degree	N/A		4 (20)		4 (10)	
	Doctoral degree	N/A		4 (20)		4 (10)	
**Parental status, n (%)**							
	Biological parent	N/A		18 (90)		18 (45)	
	Step parent	N/A		0 (0)		0 (0)	
	Adoptive parent	N/A		2 (10)		2 (5)	
**Diagnosed condition, n (%)**							
	Obsessive-compulsive disorder	4 (20)		N/A		4 (10)	
	Autism	5 (25)		N/A		5 (13)	
	Attention-deficit/hyperactivity disorder	3 (15)		N/A		3 (8)	
	Anxiety	4 (20)		N/A		4 (10)	
	Tourette	1 (5)		N/A		1 (3)	
	No clinical diagnosis or symptoms	9 (45)		N/A		9 (23)	
Age, mean (SD)			14.9 (2.2)		48.3 (6.4)		N/A

^a^Values may not total to 100 due to categorical overlap (eg, comorbidities).

^b^N/A: not applicable.

### Data Collection

**Figure 1:**
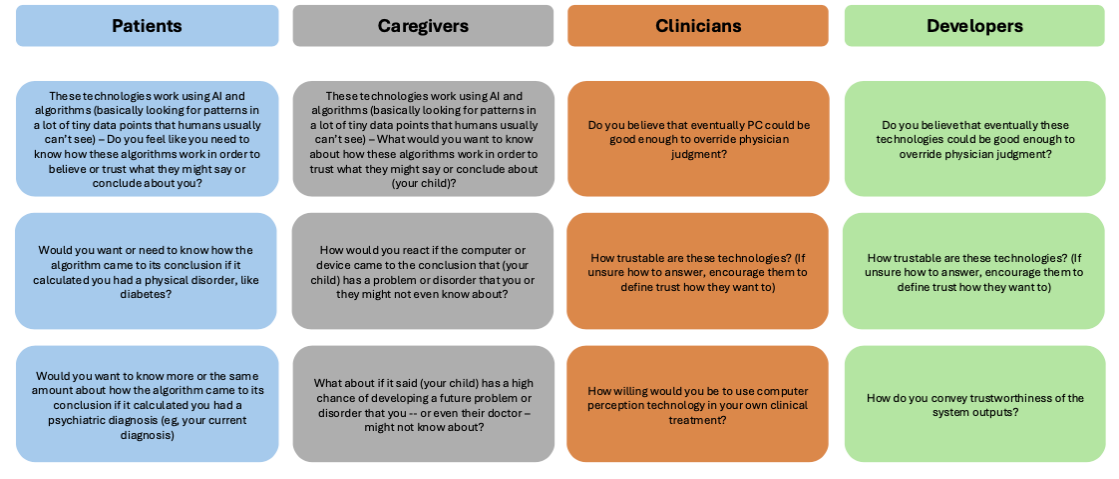
Interview guide questions across stakeholder groups. AI: artificial intelligence.

Participants were interviewed between January and August 2023 by a master’s-level (MEH) and a post-baccalaureate-level (AS) research assistant with extensive training in qualitative interviewing and analysis. Interviews were semistructured and broadly designed to explore perspectives on the potential benefits, risks, and concerns surrounding the integration of CP technologies into clinical care. Separate but parallel interview guides were developed for each stakeholder group, with the same constructs explored across groups. A subset of questions focused on perceived trustworthiness of CP systems and rationales and contingencies for trust (Figure 1). All questions were reviewed by our team of experienced bioethicists and mental health experts. Initial drafts of the interview guides were piloted with 2 psychologists (EAS and CJZ) specializing in adolescent mental health, resulting in minor clarifications to wording. Interviews lasted an average of approximately 45 minutes. Recruitment continued until we observed significantly diminished novel informational returns from each subsequent interview (saturation), which occurred at around 20 interviews per stakeholder group. All participants were invited via email to complete a brief survey to gather the demographic information presented in Table 1.

### Ethical Considerations

This study was reviewed and approved by the Baylor College of Medicine Institutional Review Board (approval number H-52227), which waived the requirement for written consent because the research procedures (interviews, deidentification of transcripts, and storage on secure servers) involved minimal risk to participating stakeholders. Thus, participants provided verbal consent. Minors provided assent with parental consent. Interviews were conducted via a secure video conferencing platform (Zoom for Healthcare; Zoom Communications, Inc), were audio-recorded, transcribed verbatim using a secure vendor, and subsequently deidentified for analysis. All results are reported in aggregate and are not linked to any identifiable participants, including in supplementary documents. Participants received US $50 gift cards for their time.

### Data Analysis

Interviews were analyzed using MAXQDA software (VERBI GmbH). Led by a qualitative methods expert (KKQ), team members (AS and MEH) developed a codebook to identify thematic patterns in stakeholder responses to the topics outlined above. Each interview was coded by merging the work of 2 independent coders (AS and MEH) to reduce interpretive bias and enhance reliability. Transcripts were coded inductively using thematic content analysis [30,31], which entailed progressive abstraction of relevant quotations. This process involved reading every quotation to which a given code was attributed, paraphrasing each quotation (primary abstraction), further identifying which constructs were addressed by each quotation (secondary abstraction), and organizing constructs into themes. To enhance the validity of our findings, all abstractions were validated by at least one other member of the research team. We then calculated the number of respondents who discussed each theme to gauge the proportion of stakeholders raising or engaging with each concern or theme. In rare cases where abstractions reflected differing interpretations, members of the research team met to reach consensus. We explored potential differences in perspectives across stakeholder groups as well as within groups (eg, by diagnostic condition among patients). Specifically, we identified salient themes across stakeholder groups using the strategies outlined above and examined, both quantitatively and qualitatively, any disproportionate representation of particular stakeholders within each theme. Frequencies and percentages are reported as descriptive rather than inferential statistics and are therefore not intended to suggest any level of statistical significance.

## Results

### Overview

Stakeholders (whose demographics can be found in Tables 1 and 2) identified 5 key criteria for appraising the trustworthiness of computer-perception technology outputs (Table 3): (1) alignment with subjective experiences and established epistemologies; (2) evidence of rigorous training and validation; (3) output explainability, including clear visibility into the processes, variables, and criteria driving AI conclusions; (4) capacity to address the complexity and diversity of illness; and (5) the role of AI outputs as enhancing—not replacing—human expertise.

**Table 2:** Demographics for interviewed clinicians and developers.

Demographics		Clinicians (n=20)	Developers (n=21)	Total (N=42)
**Gender, n (%)** ^a^				
	Male	10 (50)	18 (86)	28 (67)
	Female	10 (50)	3 (14)	13 (31)
**Profession, n (%)**				
	Clinician	3 (15)	N/A^b^	3 (7)
	Clinician-researcher	14 (70)	N/A	14 (33)
	Clinician-developer	3 (15)	4 (19)	7 (17)
	Developer	N/A	17 (81)	17 (40)
**Specialty, n (%)**				
	Psychiatry	7 (35)	N/A	7 (17)
	Psychology	7 (35)	N/A	7 (17)
	Neuroscience	4 (20)	N/A	4 (10)
	Industry	N/A	15 (71)	15 (36)
	Academic	N/A	3 (14)	3 (7)
	Cross-sector	N/A	3 (14)	3 (7)
	Other	2 (10)	N/A	2 (5)

^a^Values may not total to 100% due to categorical overlap.

^b^N/A: not applicable.

**Table 3:** Emerging criteria for trustworthiness across stakeholder groups.

Computer perception outputs are trustworthy if they...		Developers (n=20)	Clinicians (n=20)	Adolescents (n=20)	Caregivers (n=20)	Total (N=80)
**Align with existing epistemologies, n (%)**						
	Align with existing beliefs, perspectives, and experiences	3 (15)	6 (30)	7 (35)	1 (5)	17 (21)
	Align with other trusted sources of information	1 (5)	8 (40)	5 (25)	8 (40)	22 (28)
**Result from rigorously trained and validated models, n (%)**						
	Provide evidence of transparent, representative, and extensive training approaches	3 (15)	5 (25)	0 (0)	5 (25)	13 (16)
	Provide evidence of accuracy, consistency, and robust validation	6 (30)	7 (35)	3 (15)	3 (15)	19 (24)
**Are explainable, n (%)**						
	Enable visibility into variables contributing to outputs	0 (0)	1 (5)	2 (10)	4 (20)	7 (9)
	Convey clear classification criteria	2 (10)	6 (30)	1 (5)	2 (10)	11 (14)
**Account for complexity and diversity of conditions, n (%)**						
	Convey ability to account for complexities of illness	4 (20)	5 (25)	0 (0)	1 (5)	10 (13)
	Address heterogeneity in patient symptom experience	1 (5)	0 (0)	1 (5)	1 (5)	3 (4)
Are used as to support, rather than replace, clinician judgment, n (%)		2 (10)	5 (25)	0 (0)	5 (25)	12 (15)

### Theme 1: Alignment With Established Epistemologies

#### Alignment With Subjective Experiences

Stakeholders consistently highlighted that the trustworthiness of CP technologies hinges substantially on the alignment of outputs with a user’s subjective experiences (Textbox 1). They repeatedly emphasized the irreplaceable role that human insight and subjective understanding play in health care decision-making, suggesting that trust increases when AI-generated insights confirm or reflect an individual’s own feelings or perceptions. Patients described a heightened sense of confidence in these tools when outputs resonate directly with how they actually feel. We did not find meaningful nuances in perspectives between patients with differing conditions.

**Theme 1 illustrative quotes: aligns with established epistemologies.**

1. Alignment with subjective experiences

- “There’s nothing better than what a human could do for another human to help them, because humans know practically what’s inside of them. And they have experience in that level.” [Patient, P_09]
- “Well...if I realized that I was starting to feel a way that it [the output] said I was, then I would just feel like I would latch onto the idea more. It wasn’t there before, but now I’m thinking about it.” [Patient, P_14]
- “If it says one day it thinks that you’re very sad, but you don’t think you’re sad...I’m not sure [that I would trust it]. Maybe?” [Patient, P_01]
- “If my doctor also believed that and he helped me see the signs and led me to, ‘This is why it’s this way.’ But if the computer only told me that and they didn’t help me give reasons as to why it was that way, I don’t think I would really trust it.” [Patient, P_17]
- “Well, I guess I would probably say that it’s based on millions of people’s data. I said it gives us some estimate, but obviously it doesn’t say about your subjective experience, but it’s something that we can consider in making sense of your problems.” [Clinician, C_02]
- “The objective measures are too nonspecific...Someone can perceive they are not sleeping well, but then objectively, wait, you’re sleeping nine hours...there’s a disconnect there. The quality, for example, in sleep, it’s not just quantity...It’s the quality. How does someone feel? So I think that will always be important in psychology, study of cognitions, behavior, feelings.” [Clinician, C_14]
- “I don’t really know. I mean, I guess I need to understand how the data was collected and how it’s processed and what was it actually looking at and how was it interpreting that....On the last study that [my son] participated in some of his answers, some of the assessment in the report were really different from what I thought...So I was like...curious about, okay, this is kind of my assumption and this data shows it’s something really, really different, I would want to understand that.” [Caregiver, CG_01]

1. Alignment with trusted sources

- “I wouldn’t just take its word for that. I definitely want to see my doctor and run tests and things before that was the end-all-be-all.” [Patient, P_17]
- “I think it would also help me to have clinical applications. So if Dr. [Redacted] was like, ‘Yeah, we’re using this as part of our assessment now, and here’s how it works...,’ I think if people that I trust and respect were using it, that would help in addition to the empirical stuff.” [Clinician, C_15]
- “With my patients, if I’m recommending something that came from Google or Amazon or heaven forbid, Meta, they’re reluctant but they ask questions. But if I recommend something that was developed by the VA, I get no questions.” [Clinician, C_20]
- “I would then try to get some human expertise on it. I need to talk to a doctor. I need to go get this further looked into, because I’m never going to allow something to tell me something about myself or my child that I’m not going to get a second opinion on. So yeah, it’s, ‘Okay computer, thanks, but I’ll take him to the doctor and we’ll see.’ And if this second opinion or third opinion comes to the same conclusion, then I’m like, ‘Okay, I can believe that.’” [Caregiver, CG_08]

Several participants also emphasized the mediating role of clinicians in contextualizing these subjective alignments. One patient noted that trust in CP technologies depends not only on the system’s insights but also on a clinician’s ability to connect those findings to the patient’s unique circumstances. Clinicians and developers similarly recognized that subjective perspectives remain the primary source of insight into a patient’s condition, with one clinician discussing the interplay between objective metrics and subjective perceptions. Another clinician stressed the importance of remembering that CP tools—even if based on “millions of people’s data”—still do not capture an individual’s subjective experience and must be considered in tandem with the person’s own account.

#### Alignment With Trusted Sources

Participants also highlighted the importance of AI diagnostic tools conforming to trusted clinical frameworks and evidence-based practices. Clinicians emphasized that trust is reinforced when tools align with established diagnostic criteria, such as the Diagnostic and Statistical Manual of Mental Disorders (DSM), rather than introducing novel or unvetted paradigms. They cautioned that deviating from known models could undermine confidence in the technology.

The credibility gained from respected professionals endorsing CP tools was a critical factor for many participants. Some clinicians noted that endorsements by “people that I trust” can increase their receptivity to the technology. Patients echoed these sentiments, expressing a preference for verifying any AI-generated results with a trusted clinician before accepting them as final. Similarly, the development of CP tools by publicly funded institutions was viewed as a mark of transparency and openness, making patients and clinicians more inclined to trust them compared with tools developed by private industry.

### Theme 2: Rigorous Training and Validation

#### Evidence of Transparent, Representative, and Extensive Training Approaches

Stakeholders emphasized that trust in AI diagnostic tools relies on robust training processes (Textbox 2). Many stressed the need for representative datasets that reflect diverse populations and conditions to ensure that CP tools can learn from sufficiently extensive samples. Clinicians, for instance, raised concerns about the reliability of outputs when no gold standards exist across different measurement devices. They noted that variation in data collection methods and population norms can impact both reliability and validity.

**Theme 2 illustrative quotes: evidence of rigorous training and validation of models.**

**1. Evidence of transparent, representative, and extensive training approaches**

- “Steps, heart rate, all that stuff varies widely among different types of actigraphs...What is the gold standard? How do we determine that? Do we have to norm that for every population, or is that understood and then we can just deploy these devices?” [Clinician-developer, CD_02]
- “We have such small cohorts...it’s definitely not ready for a diagnostic tool. But I think we’re laying the groundwork to move toward that point though.” [Developer, D_10]

**2. Evidence of accuracy, consistency, and robust validation**

- “Yeah, so if the output said something you don’t believe, maybe doing more accurate testing about whatever.” [Patient, P_11]
- “First collect enough data that you see dependability, validity, and that you can see change and that you can predictably track outcomes...and be able to say, ‘Yep, it was correct and that it made a difference over what would have happened otherwise.’” [Clinician, C_01]
- “It really relies on both the training datasets they’re using and then really that validation that they can do on a recognized cohort. So absent of seeing that, I couldn’t really say.” [Caregiver, CG_09]
- “This is the other point. The FDA, I think where it comes to regulations, has also to start dipping. We probably have some approved algorithms that you could scrutinize, and maybe the approval should be withdrawn at some point. If you are, for instance, a Black woman coming with a breast cancer problem to an oncologist, you want to trust a model that’s really trained on 95% of white women? Probably not.” [Developer, D_01]

Skepticism also emerged regarding the current readiness of CP tools for diagnostic use. Participants pointed out that small cohorts or nonrepresentative samples remain significant barriers to achieving the reliability required for clinical deployment. Several further noted that when datasets fail to capture individuals’ unique needs or contexts, the resulting recommendations may be poorly aligned with a person’s actual interests or circumstances.

#### Evidence of Accuracy, Consistency, and Robust Validation

Stakeholders underscored the foundational role of transparent and robust validation processes in establishing trust in CP tools. They highlighted the need for clear evidence demonstrating accuracy, consistency, and clinical reliability. Clinicians, in particular, emphasized the importance of rigorous, longer-term studies to confirm that these tools are dependable and offer added value over established approaches for identifying and addressing illness. Caregivers expressed a desire to see validation “on a recognized cohort,” and several participants cautioned that models trained on large, generalized datasets may fail to capture the nuances of individual differences.

Patients similarly noted that they would need additional evidence or follow-up testing if AI outputs contradicted their own perceptions. Some participants likened the evaluation of these technologies to regulatory processes, suggesting that frameworks similar to how “an FDA regulates a new drug” (eg, safety and efficacy testing) could be appropriate for AI systems used in higher-stakes contexts. Across the board, respondents reiterated that trust in CP tools depends on demonstrated performance and reliability under real-world conditions.

### Theme 3: Outputs Are Explainable

#### Enable Visibility Into Variables Contributing to Outputs

Stakeholders emphasized the importance of transparency and explainability in AI-generated diagnostic outputs as a critical foundation for trust and effective clinical decision-making (Textbox 3). Clinicians noted that understanding the variables and data informing AI outputs is essential for interpreting them accurately and integrating them into practice. Patients echoed this need for transparency, expressing a desire to access both raw data and clear summaries to better understand how diagnostic conclusions were reached, even if they might not fully grasp every technical detail.

**Theme 3 illustrative quotes: outputs are explainable.**

**1. Enables visibility into variables contributing to outputs**

- “I might not understand the raw [data], but I’m going to figure it out. And if you explain it [the outputs] to me...I need to see, I’m very much about, prove it to me. Show me. Just because this says it, doesn’t mean I’m going to believe it. You’ve got to show me how and why you came up with this.” [Caregiver, CG_08]

**2. Conveys clear classification criteria**

- “What are the criteria? What are the cutoffs? How is the conclusion being made?...If they’re just using something that’s established like DSM criteria, that’s cool. But if it’s something that’s created based on the computing thing, then I’d wonder how did you get that? Why is the cutoff 15 and not 20?” [Clinician, C_15]
- “I don’t think that many of us, even who are highly educated or in health care, really understand how these things work.” [Clinician, C_20]
- “In terms of clinical utility, I think it would be extremely helpful to understand an algorithm’s rationale...Because if I think X, even though I have clinical experience, and the algorithm says Y, and I go, ‘Okay, why do you think that?’ and it goes, ‘Because of X, Y, and Z,’ I should be able to understand...And if you completely disagree with an algorithm, and you think its rationale is completely illogical, I think you could override it.” [Caregiver, CG_10]

Several participants noted that when AI-generated outputs appear counterintuitive, detailed explanations can help provide clarity and reassurance. This need for explainability extended across diagnostic contexts, with some caregivers emphasizing that any condition—physical or psychiatric—should be accompanied by sufficient transparency about how the system arrived at its conclusion.

#### Convey a Clear Classification Criteria

Stakeholders also highlighted the necessity of clear, logical explanations for how CP systems classify data. Many participants emphasized that understanding the algorithm’s rationale enables them to make more informed decisions, particularly when recommendations diverge from their own impressions. Transparent explanations were widely viewed as essential to ensuring the clinical utility of these tools.

While some participants acknowledged that not all users would fully grasp the technical complexities of CP algorithms, they emphasized the need for an accessible “basic understanding” of how decisions are made. Clinicians highlighted the importance of knowing the thresholds and criteria underlying system outputs, including why particular cutoffs are selected. They stressed that AI-generated classifications should align with established standards, such as the DSM, cautioning that reliance on novel or proprietary criteria—especially without clear justification—could undermine trust in the technology.

### Theme 4: Outputs Demonstrate Capacity to Account for Complexity and Diversity

#### Convey Ability to Account for the Complexities of Illness

Stakeholders repeatedly pointed out the challenges of applying CP tools to the nuanced and multifaceted domain of mental and emotional health (Textbox 4). One clinician noted that while the technology may capture “basic” emotions such as anger or happiness, it is likely to struggle with more complex or mixed emotional states. Others emphasized that mental health exists along a continuum, making it difficult for AI systems to assess using rigid or discrete measures. Clinicians and caregivers also questioned whether these tools can adequately capture the subtleties of a child’s or patient’s behavior, noting that individual data points often lack the contextual information needed for accurate interpretation. Across all groups, stakeholders cautioned against treating CP systems as definitive diagnostic instruments, given the complexity of mental and emotional health and the high potential for misinterpretation.

**Theme 4 illustrative quotes: conveys complexity and diversity of illness.**

**1. Accounts for the complexities of illness**

- “What scares me about it is that these tools will be used as if they’re perfect...In the behavioral realm, we’re much likelier to be further away from perfect accuracy than you are when you’re trying to detect cancer or something.” [Clinician, C_05]
- “I think we’re a little too high dimensional for all of that...You can tell if somebody’s angry. You can tell if somebody’s happy...But there’s a lot of combinations of these things that we’re not able to get.” [Clinician, C_10]
- “Even clinicians can’t be a hundred percent in agreement if someone’s on the spectrum or off the spectrum....That’s definitely my impression, that this [computer perception technology] will never be a hundred percent accurate.” [Developer, D_08]

**2. Addresses heterogeneity in patient symptoms**

- “A physical one [condition]...if there are numbers that you need to reach to either be healthy or have the disease or whatever, versus anxiety and depression...are on a spectrum, and I think they [these conditions] are still being understood.” [Clinician, C_12]
- “This algorithm was trained on thousands of records...But even if the record quality is good, it can still probably lead you on a wrong path....I still, at least at this stage, wouldn’t trust picking out one specific patient and say, ‘Okay, that’s how this patient must be.’” [Clinician developer, CD_18]
- “The way diagnoses are set up...right now they’re categorical, even though that’s not true, and there’s this continuous spectrum. So when we do that, we get 10 diagnoses for somebody because the symptoms overlap so much. It’s not 10 diagnoses, it’s two or three.” [Caregiver, CG_06]

#### Address Heterogeneity in Patient Symptom Illness

Numerous stakeholders emphasized the overlapping nature of many medical and mental health conditions. Many expressed that they would place greater trust in tools capable of accurately identifying and representing these overlaps, as well as addressing the substantial heterogeneity in patient symptoms. Clinicians and developers noted that even large datasets may fall short in producing accurate, individualized characterizations or predictions—particularly for neurodevelopmental and psychiatric conditions whose underlying mechanisms remain poorly understood, even among experts.

Some participants stressed that diagnostic tools should be purposefully tailored to the specific complexities of individual conditions rather than relying on broad, generalized data-collection approaches. Biometrics such as heart rate variability were described as “nonspecific”—useful for signaling general abnormalities but insufficiently precise for identifying a particular diagnosis. Clinicians and developers emphasized that unless these systems are carefully calibrated to account for the distinct features of each condition, their outputs risk lacking the specificity needed for meaningful clinical use.

### Theme 5: AI Outputs As Supplemental Tools to Support Clinician Judgment

Participants indicated that trust in AI diagnostic tools would be highest when CP systems function as supportive aids rather than replacements for human expertise (Textbox 5). Clinicians, caregivers, and developers agreed that these tools should enhance—rather than supplant—clinical decision-making. One clinician described CP technologies as “an additional piece of information,” while emphasizing that face-to-face relationships remain central to health care. Caregivers similarly compared algorithmic results to routine diagnostic tools such as scans, noting that while these outputs can be valuable, they should not overshadow the clinician’s own judgment.

**Theme 5: used as supplements to support clinician judgment.**

**1. ...Aids, not replaces, existing diagnostic approaches...Is not sole basis for evaluation**

“I feel like my doctors should take into account what the monitors have to say but also have a perspective on who I am and not just what the numbers are saying...it should be just one component of the overall assessment.” [Patient, P_10]

“I wouldn’t just take its word for that. I definitely want to see my doctor and run tests and things before that was the end-all-be-all.” [Patient, P_17]

“I definitely think it could be a tool that would be an additional piece of information, but I always think having a good relationship with your doctor, the in-person interaction, is probably primary and this would be a helping tool. Hopefully, it’s not the only diagnostic tool.” [Clinician, C_11]

“I would want them [clinicians] to be interested in using it as another tool but not relying solely on it.” [Caregiver, CG_09]

“It [the clinical decision] should always be the clinician’s judgment. Today, a clinician uses a CAT scan, a PET scan, an MRI to measure something in the brain...They’re not blindly following what the image shows. If they can sense that something’s off with what their technology is providing them, it’s always their own judgment whether or not they want to move forward with an action.” [Caregiver, CG_14]

A number of participants expressed skepticism about the current capabilities of CP tools, particularly for mental health diagnostics. Developers questioned whether wearable data alone could reliably support differential diagnoses, noting that “we haven’t gotten to [that] point” clinically. Another developer emphasized the challenge of achieving diagnostic consensus even among trained professionals, suggesting that CP tools could compound—rather than resolve—existing uncertainties, especially if clinicians interpret CP outputs through the lens of their own assumptions or biases.

Caregivers also raised concerns about over-reliance on CP tools, particularly among populations less familiar or comfortable with these technologies, who might place greater trust in human judgment. Some suggested presenting CP findings as “supporting information,” rather than definitive conclusions, to help build confidence in their use. Ultimately, many stakeholders emphasized that fostering trust in CP systems requires demonstrating transparency, reliability, and a clear commitment that human expertise remains the final authority.

## Discussion

### Principal Findings

As CP technologies become increasingly integrated into clinical practice, questions surrounding their trustworthiness remain central to their adoption and utility. Several themes emerging from our interviews reflect well-established concerns—namely, the need for rigorous and representative model training, explainable outputs, and the use of CP tools to augment rather than replace human judgment [5,6]. However, 2 findings stand out as novel and carry important implications for implementation.

One of the most consequential findings is that stakeholders were more inclined to trust CP systems when their outputs aligned with existing assumptions, clinical impressions, and validated assessment tools. This tendency was especially pronounced among clinicians and caregivers, whose fiduciary and caretaking responsibilities may heighten the motivation to verify CP outputs against trusted sources of information or firmly held intuitions. Our data suggest that trust in machine-generated inferences is not solely—or even primarily—a function of a system’s performance, processes, and purpose, as some scholars have proposed [6], nor is it driven chiefly by concerns about impacts on the humanistic dimensions of care [22,29]. Rather, trust is shaped by the degree to which CP outputs resonate with established medical knowledge, clinical reasoning, and patients’ subjective experiences. Clinicians suggested that they would place greater trust in outputs that align with their own clinical impressions, while adolescents reported being more trusting of inferences that corroborate their subjective experiences and more inclined to question those that did not. Many caregivers similarly noted that they would trust outputs that confirmed their understanding of their child’s mental health. Respondents across groups also emphasized that they would place greater confidence in outputs that were consistent with insights from other trusted sources—such as their own clinician or professional colleagues—or that were produced by tools developed through government- rather than privately funded research [27,28].

### Epistemic Alignment As a Core Driver of Trust

This finding—that people tend to trust CP systems when outputs confirm their existing epistemologies—is one of the most compelling and cautionary takeaways of this investigation. On the one hand, alignment with existing impressions or understandings can function as a valuable plausibility check for CP systems whose accuracy, validity, reliability, and other performance metrics remain imperfect. When outputs appear too far afield or sharply contradict established understandings, low trust may be appropriate. Consistent with prevailing recommendations that CP and other AI-based tools should augment rather than replace human judgment, participants described using alignment with their own instincts (eg, clinical judgment or subjective experience) or with trusted sources of information (eg, colleagues’ perspectives or scientific consensus) as a way to corroborate a system’s conclusions. Many felt reassured when a system “got it right,” particularly when it echoed adolescents’ internal states or matched clinicians’ diagnostic expectations. In this sense, alignment can help calibrate early trust in emerging technologies and offer a form of intuitive verification.

However, this same dynamic introduces real risks, particularly for clinicians seeking to implement CP tools. Alignment with one’s expectations does not guarantee that a model’s outputs are accurate. People—including highly trained clinicians—are vulnerable to cognitive biases that make them more likely to trust information that confirms what they already believe and to discount information that challenges those beliefs. The most relevant is confirmation bias, the well-documented tendency to seek, interpret, and remember information in ways that reinforce prior assumptions [32]. Work in cognitive and decision sciences similarly shows that individuals tend to favor information that aligns with existing knowledge or expectations [33,34], including through anchoring effects that skew judgments toward an initial impression [35]. In clinical practice, these biases can lead stakeholders to embrace CP outputs that affirm their expectations and dismiss those that contradict them [36,37]. Rather than expanding diagnostic reasoning, CP systems optimized for user trust may inadvertently narrow the scope of information users consider, reinforcing existing assumptions and contributing to epistemic echo chambers [38].

This tension reveals a deeper paradox at the heart of CP integration. CP systems are designed to uncover patterns and associations that may not be readily accessible to human cognition, yet their trustworthiness is often judged by whether their outputs *already make sense* to users. A core rationale for developing CP is precisely its capacity to detect subtle indicators of pathology or complex, multivariate patterns that humans might overlook. Distrusting these systems because they begin to perceive things that users do not—or cannot—introduces a fundamental conflict between innovation (challenging existing paradigms) and epistemic alignment (conforming to them). This paradox risks undermining the very purpose and promise of CP systems: to extend, rather than merely reproduce, human ways of knowing.

Relatedly, CP tools have been slow to integrate with traditional categorical diagnostic systems such as the DSM and the International Classification of Diseases. This is due in part to the fact that many CP tools were developed in response to longstanding criticisms of these nosologies, which some argue have not substantially advanced understanding or treatment of mental health conditions conceived as discrete categories for more than a century. Computational approaches in psychiatry emerged alongside newer paradigms—most notably the Research Domain Criteria [38] and the Hierarchical Taxonomy of Psychopathology [39]—which aim to reconceptualize psychopathology using dimensional frameworks grounded in empirically observed, overlapping, and sometimes hierarchical symptom patterns. As these dimensional frameworks remain largely investigational and have not yet been broadly adopted in clinical practice, the tools developed to support them (including CP technologies) are subject to heightened scrutiny and, at times, skepticism. Clinicians may consequently discount these tools, limiting opportunities to incorporate perspectives that extend beyond established nosological frameworks and potentially contributing to continued stagnation within psychiatry and behavioral health. At the same time, developers seeking greater acceptability and uptake may feel compelled to design systems that conform to rather than challenge prevailing clinical assumptions. The broader push toward personalized or user-adaptive AI further increases the likelihood that systems will be engineered to deliver psychologically satisfying outputs—those that feel intuitively “right” to users—rather than ones that meaningfully expand clinical understanding. Although such alignment may enhance user engagement, it carries substantial risk in high-stakes domains such as health care, where decisions must be grounded in empirically verifiable evidence and objective indicators rather than subjectively endorsed or familiar interpretations [40].

### Relational and “Distributed” Trust

Another key finding is that stakeholders favor models that are endorsed by people and institutions they already trust. This form of trust—often referred to as “surrogate” or “relational” trust—rests on the assumption that if a trusted other (eg, a clinician, colleague, institution, or scientific community) has confidence in a CP tool, then that trusted party has presumably conducted an adequate appraisal of the tool’s quality and reliability [1]. Multiple clinicians reported that they would attribute greater trustworthiness to AI models endorsed by respected institutions or medical authorities, recognizing that such endorsements typically signal more stringent oversight in model development, validation, and ongoing evaluation. One clinician noted that patients were far more willing to trust AI developed by government agencies (eg, the Veterans Affairs) than by corporate technology firms, on the grounds that government agencies demand greater accountability and are not profit-motivated. This finding offers empirical support for recent conceptual work suggesting that trust is grounded not simply in the technical features of a system, but in the human relationships and social contexts in which the system is embedded [41].

While the logic of these kinds of trust attributions—akin to the saying “a friend of a friend is my friend”—may not always be reliable, it nonetheless functions as a rational heuristic in contexts where individuals lack the time, expertise, or information needed to rigorously evaluate an unfamiliar system. Such conditions are especially common in health care, where clinicians must frequently make decisions quickly and under uncertainty. Clinicians and other stakeholders who do not have the capacity or digital literacy to critically assess the training quality, performance characteristics, or contextual relevance of specific CP outputs for each patient—which may vary substantially across metrics, populations, and clinical settings—may be particularly inclined to rely on surrogate or relational trust when determining whether and how to incorporate these tools into care [41].

An important implication is that trust in CP systems may be conferred less on the basis of their intrinsic validity and more on the perceived credibility of the people, institutions, or frameworks associated with them, raising critical questions about how such trust is earned, signaled, and potentially misplaced. To ensure that this heuristic is not exploited or misapplied, CP technologies must be embedded within institutional systems that uphold accountability, professional standards, and ethical commitments. In other words, there must be a higher threshold for implementation—a kind of institutional “entry fee”—so that not just any system can be integrated into clinical workflows. This may entail the establishment of hospital- or system-level review boards or other oversight mechanisms to vet and approve CP systems before deployment, thereby alleviating the evaluative burden placed on individual clinicians (or patients and caregivers) to independently appraise trustworthiness. In some cases, this may require an explicit acknowledgement that clinicians, patients, and other stakeholders may not have the time, resources, or skill sets to effectively vet CP and other AI-based tools in complex and fast-paced medical settings, and that trust in these tools, by extension, must be distributed institutionally, organizationally, or across medical and scientific communities in ways that enable the safe and efficient use of these systems (in much the same way that trust is distributed in banking and other financial institutions). Yet, such reforms face structural resistance from prevailing clinical paradigms that place agency, responsibility, and ultimately culpability on the shoulders of individual providers [42], potentially limiting the feasibility of more distributed or institutionally anchored models of accountability.

### Trust Calibration Through Education and User-Centered Design

If both confirmation bias and relational trust shape how CP technologies are evaluated and adopted—particularly in high-stakes, high-uncertainty settings—then it becomes even more important to help stakeholders distinguish between trust that feels “intuitive” (but may be unwarranted) and trust that is well-founded. Without adequate support, stakeholders may find it difficult to judge when trust is justified—for example, when an output is genuinely accurate, well-calibrated to the patient population, or appropriately contextualized—and when trust may instead be misplaced. At present, however, there is no consensus on what kinds of knowledge or competencies stakeholders need in order to use CP tools responsibly. Further empirical work is therefore needed to define these requirements. Certainly, training is needed to equip users with the epistemic tools to recognize and resist common cognitive distortions, such as confirmation bias and anchoring effects, that can subtly influence how AI outputs are interpreted. However, the solution likely extends beyond simply increasing AI literacy. As Kostick-Quenet and Gerke [37] argue, mitigation of biases (of all sorts, including but not limited to oft-cited algorithmic racial biases) must be addressed not only through education but also through intentional design [37]. Drawing on decision science and behavioral economics, they call for embedding trust-calibration mechanisms into the design of AI interfaces themselves—such as nudges that interact with the user, prompting them to question outputs or consider alternative explanations. Through consistent communication within the context of a user’s needs, goals, and feedback, these interventions can foster reflective rather than passive engagement with AI-generated insights.

For instance, CP systems could include interface prompts that encourage clinicians to document the factors—beyond the algorithm’s recommendation—that shaped their clinical judgment, or to reflect on situations in which the system’s suggestion may be flawed or inapplicable. Such design features can help shift trust away from automatic acceptance based on familiarity or perceived alignment and toward a more reflective, evidence-informed mode of engagement. Additional interactive elements should also be systematically evaluated for their ability to promote deeper critical reflection. These elements should not only prompt clinicians to appraise the validity of system outputs but also to question entrenched assumptions, consider alternative clinical perspectives, and use CP-derived insights to reframe their understanding of patients’ illness experiences and care needs.

### Limitations

Our efforts to sample broadly across sociocultural and demographic groups are reflected in the diversity of our participant sample. Nevertheless, because most adolescent and caregiver participants were recruited from a single geographical region, our findings may have limited generalizability to other regions of the country. Additionally, because interviews were conducted in English, perspectives from non-English speakers may be underrepresented. We also did not exhaustively examine potential variation by gender, race, ethnicity, or other sociodemographic factors such as digital literacy, largely because our goal was to identify a comprehensive range of trust considerations rather than to explain their variability. This formative work is intended to inform more systematic examinations of distinct sources of variation using a structured survey (a subsequent phase of this research). Lastly, all findings reflect not only themes that emerged from the interviews but also our research team’s interpretation of them, shaped by our own perspectives and biases.

### Conclusion

This study advances empirical understanding of how trust is formed and calibrated around AI-based CP technologies. Our findings reveal that trust is not simply a function of technical performance, transparency, or utility, but is significantly shaped by epistemic alignment—the degree to which CP outputs affirm users’ preexisting beliefs, experiences, or clinical frameworks. Such alignment can help users intuitively verify outputs in uncertain clinical environments, but also risks reinforcing confirmation bias and overreliance on surrogate or relational trust (trust based on endorsement from familiar individuals or institutions rather than on rigorous, independent vetting). These dynamics likely reflect adaptive cognitive strategies for assessing novel tools in high-stakes situations characterized by substantial uncertainty. However, they raise concerns that CP systems may garner high levels of trust only when they echo users’ existing assumptions or reflect consensus understandings, rather than offering novel information intended to expand users’ perspectives.

To address these concerns, we argue that CP tools should be embedded within institutional environments that uphold accountability, ethical integrity, and rigorous review before deployment, thereby easing the burden on individual stakeholders to independently evaluate trustworthiness without the necessary resources, background knowledge, or skill sets. Second, we propose that educational initiatives move beyond calls for AI literacy to emphasize design-based interventions that help users distinguish between intuitive and well-founded trust. Beyond awareness training in cognitive bias and critical appraisal, further research should seek to identify user interface features—such as prompts that encourage counterfactual reasoning and contextual scrutiny—that foster reflection and support thoughtful engagement with CP outputs. These strategies may help address a key paradox: CP tools must align with existing clinical reasoning paradigms to gain trust, yet must also challenge them in order to advance and improve care.

## Appendix

Multimedia Appendix 1 Consolidated Criteria for Reporting Qualitative Research (COREQ) checklist.

## Abbreviations

AI: artificial intelligence
COREQ: Consolidated Criteria for Reporting Qualitative Research
CP: computer perception
DSM: Diagnostic and Statistical Manual of Mental Disorders

## Data Availability

The datasets presented in this article are not readily available because full datasets must remain unavailable to ensure the deidentification of interview participants. Requests to access the datasets should be directed to KKQ.
